# Pediatric Perioperative Pulmonary Arterial Hypertension: A Case-Based Primer

**DOI:** 10.3390/children4100092

**Published:** 2017-10-24

**Authors:** Shilpa Shah, Jacqueline R. Szmuszkovicz

**Affiliations:** 1 Children’s Hospital Los Angeles, Division of Anesthesia and Critical Care Medicine, Los Angeles, CA 90027, USA; shishah@chla.usc.edu; 2 Keck School of Medicine, University of Southern California, Los Angeles, CA 90033, USA; 3 Children’s Hospital Los Angeles, Division of Cardiology, Los Angeles, CA 90027, USA

**Keywords:** pediatric pulmonary hypertension, perioperative management, pulmonary hypertensive crisis

## Abstract

The perioperative period is an extremely tenuous time for the pediatric patient with pulmonary arterial hypertension. This article will discuss a multidisciplinary approach to preoperative planning, the importance of early identification of pulmonary hypertensive crises, and practical strategies for postoperative management for this unique group of children.

## 1. Introduction

The perioperative period is an extremely tenuous time for patients with idiopathic pulmonary arterial hypertension (IPAH). Careful preoperative planning and meticulous postoperative management can mitigate the risks for this fragile group of patients. Postoperative management includes anticipation of pulmonary hypertensive complications, early recognition of the salient issues and prompt management.

This article will discuss an approach to the management of pediatric pulmonary arterial hypertension in the perioperative period.

## 2. Preoperative Planning

The American Heart Association (AHA)/American Thoracic Society (ATS) pediatric pulmonary hypertension guidelines emphasize the importance of preoperative planning for this high-risk group of patients [1]. A multidisciplinary approach is critical to this endeavor. The conversation should include the pediatric cardiologist specializing in pulmonary hypertension (PH), the pediatric PH nurse specialist, the cardiac anesthesiologist, the physician performing the procedure, the pharmacist and the intensive care unit staff.

The multidisciplinary team can provide suggestions for optimization of the preoperative physiologic state. This may include evaluation and treatment of comorbidities, optimizing fluid balance, optimizing right ventricular function, and adjusting pulmonary vasodilator management. If the procedure is elective, the team may suggest delaying the procedure until medical interventions to optimize the patient’s overall condition are performed. In some cases, the team may advise to not proceed with an elective surgery at all, after thorough multidisciplinary discussion of the risks and benefits.

The cardiac anesthesiologist’s role on the team is to provide a plan for intraoperative pain and sedation management, and suggested approaches to avoid arrhythmias. Hypotension or direct myocardial suppression can occur if the anesthetic agent chosen is not optimal [2]. The induction of anesthesia itself can precipitate a pulmonary hypertensive crisis. Adequate sedation must be established prior to direct laryngoscopy, as sympathetic stimulation can increase pulmonary vascular resistance [3]. The risk of intubation thus includes the effect of the necessary premedications that may cause a change in systemic vascular tone [4]. The most experienced operator should perform the intubation as the consequences of a right mainstem or esophageal intubation can be disastrous, and any length of time that the patient is not being adequately ventilated can contribute to hypercarbia, hypoxemia and acidosis and thus also precipitate a crisis. The anesthesiologist is also responsible for the process of emergence from anesthesia and extubation. Airway obstruction, hypoventilation, agitation and pain should all be avoided, or treated promptly if they occur.

The surgeon/medical physician performing the procedure can alert the team to special considerations for each particular surgery that may contribute to physiologic stressors. Examples of these intraoperative stressors might include patient positioning on their side, pneumoperitoneum, intentional deflation of one lung, and diaphragmatic compression.

The discussion regarding the postoperative monitoring plan should occur preoperatively and should include all members of the team: the intensive care unit (ICU) physician may recommend that an intraoperative pulmonary arterial catheter be placed by the anesthesiologist in a particular situation, for example [5]. They can also discuss what medications should be available in the ICU postoperatively with the pharmacist, and alert the respiratory therapy team to the need for nitric oxide.

Clearly, for patients with pulmonary hypertension, the perioperative period is a tenuous time. Benjamin Franklin is credited with saying “an ounce of prevention is worth a pound of cure”. In the case of pediatric idiopathic pulmonary arterial hypertension (IPAH) and postoperative management, perhaps it could be restated: “an ounce of prevention is worth twenty pounds of cure”.

First, we will present a hypothetical case (based on multiple real cases) with a suboptimal outcome to prompt thinking about pathophysiology, management and pitfalls to avoid.

## 3. Case

A six-year-old patient from an outside hospital comes in for extensive dental work under anesthesia. The PH team has no records on the patient and was not involved with the preoperative planning. The parents state that the patient has severe IPAH and does not have an atrial septal defect. They do not know if his right ventricular function is preserved or decreased. He is on inhaled treprostinil, oral sildenafil and bosentan at home. The parents were told that he needed to be nil per os (NPO) prior to the surgery and they did not give him his usual morning medications.

The patient was induced with propofol, intubated and then maintained on inhaled anesthetics for the duration of the case. After completion of the procedure, he was awakened and extubated in the operating room.

Intraoperatively the patient received 40 cc/kg of normal saline. The patient lands in the ICU postoperatively because the anesthesiologist notices that at the end of the case the patient has poor perfusion, systemic blood pressure 60/30 mm Hg and an oxygen saturation of 89% on 2 L per minute of supplemental oxygen delivered by nasal cannula. The patient is agitated and is complaining of pain. Over the next minutes, as a fluid bolus is being administered, he becomes unresponsive, bradycardiac and pulseless. Cardiopulmonary resuscitation is performed with chest compressions, 5 doses of epinephrine and establishment of airway with return of spontaneous circulation after 20 min. He is now intubated, has tachycardia to 140 beats per minute (bpm), is hypotensive (60/30 mm Hg) and is on an epinephrine drip of 0.2 mcg/kg/min. Echocardiogram reveals a dilated and poorly functioning right ventricle (RV) with suprasystemic RV pressures and septal deviation resulting in an underfilled and compressed left ventricle (LV).

## 4. Discussion

What are the general principles and approach to managing a patient in this situation?

### 4.1. Definitions

Pulmonary hypertension is defined as a mean pulmonary arterial pressure >25 mm Hg. Further distinction must be made between pulmonary arterial hypertension (pre-capillary) and post-capillary pulmonary hypertension—with pulmonary arterial hypertension defined as a mean pulmonary arterial pressure ≥25 mm Hg at rest, with a pulmonary arterial wedge pressure of ≤15 mm Hg and pulmonary vascular resistance index >3WUxm^2^ [6]. There are significant—and at times, even conflicting—differences in management strategies between the two entities. Here we discuss only pre-capillary pulmonary arterial hypertension.

### 4.2. Pathophysiology of Pulmonary Hypertensive Crisis

**Figure 1:**
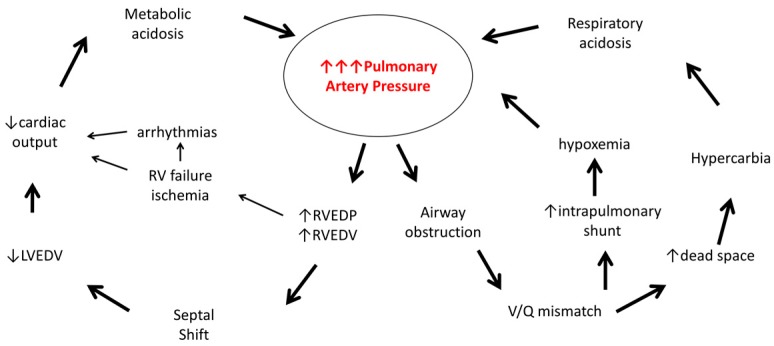
Pathophysiology of a pulmonary hypertensive crisis (Adapted from Oishi et al. [7]). RVEDP: right ventricular end diastolic pressure; RVEDV: right ventricular end diastolic volume; LVEDV: left ventricular end diastolic volume; RV: right ventricle; V/Q: ventilation/perfusion.

This child is having a pulmonary hypertensive crisis. Figure 1 illustrates the pathophysiology involved.

A trigger (hypoxemia, pain, acidosis) has caused elevation of the child’s pulmonary arterial pressure. When the pulmonary arterial pressure rises above baseline, the right ventricle, not evolved for such high afterload, has increased end diastolic pressure and end diastolic volume. In our patient, given the chronic nature of his pulmonary hypertension, it may be that the RV has some pre-existing dysfunction. Both the acuity and the degree of increased pulmonary pressures can contribute to further right ventricular dysfunction and subsequent failure. RV myocardial dysfunction and right atrial dilatation may lead to arrhythmias and worsening cardiac output. Compromised systemic perfusion and decreased coronary perfusion are the endpoints of this process.

With the right ventricular dilation, the intraventricular septum shifts towards the left and can cause compression of the left ventricle. The left ventricle, in turn, now has increased diastolic pressures and less room for filling, resulting in loss of systemic arterial output and hypotension. Because of the lack of a pop-off valve/intracardiac shunt, instead of hypoxemia, the result is hypotension.

However, that these patients have no difficulty oxygenating is not fully true. In addition to the cardiac consequences described above, ventilation can be impaired—sometimes dramatically. Airways obstruction from distention of hypertensive arterioles may lead to quite impressive changes in compliance. This acquired lung pathology will lead to further increases in pulmonary vascular resistance (through hypoxemia/acidosis/hypercapnia), contributing to further deterioration.

Metabolic and respiratory acidoses resulting from the above mechanisms will further worsen pulmonary vascular resistance and potentially create vicious cycles that will likely lead to deterioration and possibly even death in the absence of intervention [7].

### 4.3. Presence of an Intracardiac Shunt

Although the patient with pulmonary arterial hypertension may have varying degrees of lung disease, the most important anatomical distinction in this group of patients is the presence or absence of an intracardiac shunt. The patient in this scenario does not have a shunt lesion (for example, an atrial septal defect). It is sometimes said in this scenario “it is better to be blue than gray” because the patient without an atrial septal defect is not able to effectively pump their blood out to the lungs to get oxygenated and is in a low cardiac output state (“gray”). Patients who have the ability to “pop-off” from the high-pressure right side to the left side, are able to maintain their cardiac output at the expense of being more cyanotic [8,9]. The presence or absence of an intracardiac shunt lesion is thus an important part of the preoperative assessment. For completeness, we must mention that the contribution of a large intracardiac shunt to the development of pulmonary hypertension is also an important topic, but beyond the scope of this article. 

### 4.4. Management Points

#### 4.4.1. Diagnosis

Diagnosing a pulmonary hypertensive crisis requires an index of suspicion, including knowledge of previous history, predisposing and precipitating factors. A patient with a pulmonary hypertensive crisis will have a constellation of symptoms as described above—with varying degrees of cyanosis versus systemic hypotension depending on the presence of an intracardiac shunt. Invasive monitoring, including central venous pressure monitoring, and, rarely, a pulmonary artery catheter, may assist in the diagnosis [5,10]. One would expect the central venous pressure to be elevated in a patient with normal cardiac anatomy who is suffering a pulmonary hypertensive crisis. The differential diagnosis, in addition to thinking about the broad range of causes of hypotension and hypoxemia, includes consideration of other causes of RV failure such as a large pulmonary embolism.

In addition to the clinical exam, vital signs and data from invasive monitoring, one must assess markers of oxygen delivery, presence of acidosis, and end organ function via laboratory testing including blood gas monitoring, lactic acid, and renal and hepatic function tests.

In a patient with long-standing pulmonary hypertension, an electrocardiogram (EKG) may show signs of right heart abnormalities, including atrial dilation, right axis deviation and right ventricular hypertrophy (sometimes with ST and T wave changes indicating right ventricular strain). Attention to rhythm is also important as arrhythmias may manifest. Although they tend to be rare, arrhythmias are generally very poorly tolerated in this group of patients [11]. A chest radiography may show lung disease, right heart enlargement and pulmonary artery enlargement (a prominent hilum) [12]. Perhaps the most useful non-invasive imaging tool is an echocardiogram—looking specifically at right and left ventricular size and function, ventricular septal shift, valve function and estimated pulmonary arterial pressure via estimated right ventricular systolic pressures (RVSP). Additionally, if the patient has an intracardiac shunt (e.g., atrial septal defect), the direction of flow and size of the shunt can be assessed [10].

#### 4.4.2. Pre-Operative Considerations

A major point in the discussion of this clinical case is that the preoperative planning step is totally missing from this scenario. No multidisciplinary discussion of the approach to management occurred prior to the patient going to the operating room. Perhaps this pulmonary hypertensive crisis could have been avoided with proper prior planning.

#### 4.4.3. Anesthetic Considerations

Anesthetic management of patients with pulmonary hypertension should be focused on maintaining perfusion pressure, preload, systemic vascular resistance and contractility in addition to avoiding the triggers that will increase pulmonary vascular resistance (PVR) (e.g., hypoxemia/hypercarbia, acidosis, pain, agitation). Induction should be with an agent that will maintain perfusion pressures—e.g., etomidate, ketamine (in the patient who does not have depleted adrenal stores), or high dose fentanyl. Detailed discussion of inhaled anesthetic management is largely beyond the scope of this article, but isoflurane and sevoflurane cause pulmonary vasodilation and may be part of the strategy (with consideration of the possibility of negative effect on cardiac contractility and systemic vasodilation) [13,14]. Importantly, the patient must be deeply sedated prior to direct laryngoscopy—or increased PVR may result from such stimulation. Maintenance of perfusion pressure is essential and there should be a low threshold to add on a vasopressor, as systemic hypotension can rapidly lead to compromised coronary perfusion, septal shift towards left ventricle and a further downward spiral. Consideration of starting a vasopressor prior to induction is warranted, especially if RV dysfunction is present. Fluid management and use of pulmonary vasodilators may also be indicated (see post-operative discussion below).

#### 4.4.4. Post-Operative Management

The overriding principles in the management of a pulmonary hypertensive decompensation are twofold: (1) reduction of PVR and (2) support of the RV and restoration of perfusion pressures—for both coronary and systemic organ perfusion.

##### Reducing Pulmonary Vascular Resistance: Avoiding Triggers of Vasoconstriction

Reduction of PVR includes avoiding ongoing triggers of vasoconstriction and use of targeted agents. We will first discuss triggers of vasoconstriction and then targeted agents. Acidosis, hypoxemia, excess positive end expiratory pressure (PEEP), tracheal suctioning and hypercapnia are all triggers of constriction and should all be avoided. The patient in this scenario requires immediate management of his pain and agitation/anxiety and improved oxygenation to hopefully assist in avoiding a further spiral into increasing acidosis and hypoxemia. Let us briefly discuss acidosis, hypoxemia and hypoventilation in more detail. Acidosis, whether metabolic or respiratory, causes pulmonary vasoconstriction [15]. In a pulmonary hypertensive crisis, with compromised systemic perfusion, acidosis will worsen—and the downward spiraling cycle will continue. Therefore, buffering the acidosis, addressing hypoventilation and addressing systemic cardiac output are all important measures in reversing the acidosis.

What is worse—hypercarbia or acidosis? While neither is optimal, a low pH is likely the worse culprit. There is human and animal evidence that if hypercarbia is buffered metabolically (i.e., hypercarbia with normal pH), the pulmonary vascular resistance will decrease significantly. In one study of infants after cardiac surgery, PVR was even lower in the infants after buffering of their hypercarbia compared to normal pH, normal CO_2_ state [16,17]. Rapid administration of bicarbonate can have a dramatic immediate effect on pulmonary vascular resistance. Hypoxemia leads to vasoconstriction and increased pulmonary vascular resistance, again contributing to the vicious cycle [16]. The other side of this equation is that oxygen is one of the most effective pulmonary vasodilators.

When we discuss ventilation, lung volume is another important component in pulmonary vascular tone. Both low and high lung volumes lead to increased pulmonary resistance—by compression of extra-alveolar and alveolar pulmonary vasculature respectively [18].

Thus, restoring oxygenation and ventilation is a key point in the management of these patients. The focus is on decreasing oxygen demand and improving oxygen delivery, and supplemental oxygen can be used liberally in this case. There is, in fact, even evidence that in children with pulmonary hypertension, hyperoxia itself reduces oxygen consumption [19]. Noninvasive ventilatory measures can be considered, including high flow nasal cannula oxygen and continuous positive airway pressure (CPAP), particularly in the patient with lung disease and atelectasis. However, mechanical airway pressure must be applied judiciously—high PEEP can compress alveolar capillaries and lead to increased PVR. In addition, use of high PEEP can decrease venous return and thus affect cardiac output. Judicious use of PEEP, on the contrary, can be advantageous as it can assist in efforts to prevent atelectasis and ultimately ventilation-perfusion mismatch. The goal of this approach is to reduce anatomic dead space, improve gas exchange and reduce the work of breathing.

One would prefer to avoid intubation if at all possible. However, once a patient is intubated, one must keep in mind that extubation (if associated with hypoventilation and agitation), may trigger a pulmonary hypertensive crisis as well. Timing of extubation must include consideration for optimization of ventilation and treatment of other triggers (e.g., pain) prior to proceeding with extubation.

##### Reducing PVR: Pulmonary Vasodilatory Therapy

How do pulmonary vasodilators work to help this group of patients? Vasodilators decrease afterload and thus improve right ventricular function and performance. Right ventricular wall tension is decreased, and right ventricular stroke volume is increased. When one improves the adverse right ventricular–left ventricular interaction, left ventricular filling and coronary perfusion improve, and myocardial oxygen consumption is decreased. One note of caution in the use of vasodilators: one must be vigilant about monitoring for ventilation–perfusion mismatch. Delivering more blood flow to unventilated areas of the lung may not be advantageous. In addition, one must remember that vasodilator therapies are not all pulmonary specific. A balance of systemic and pulmonary vasodilation must be carefully brokered. This is why the preferred route of administration of vasodilator therapy in crisis is inhaled, if possible. This discussion is centered around medication administration in crisis—not on chronic management (as this is a very different discussion). As mentioned above, the first direct pulmonary vasodilator administered should be oxygen—delivered effectively at 100% FiO_2_. The second drug administered is usually inhaled nitric oxide (iNO), which can lower the pulmonary vascular resistance in responsive patients, without having significant effects on the systemic vascular bed [20]. It can also improve ventilation-perfusion mismatch by selectively vasodilating ventilated lungs and therefore increasing systemic arterial saturation. Use of iNO requires monitoring of methemoglobin. Some centers also use inhaled prostacyclin analogs (e.g., epoprostenol, iloprost). These agents have demonstrated efficacy in reducing PVR in a variety of settings, but it is unclear if there are additive/synergistic effects of using both iNO and prostacyclin analogs. Of note, inhaled epoprostenol has a glycine buffering agent that has the potential to cause ventilator valve malfunction [21]. As salvage therapy for patients who have not responded adequately to iNO or prostacyclin, inhaled milrinone has been used with some effectiveness [22]. If inhaled agents are unavailable or ineffective, systemically administered vasodilators can be used. These include sildenafil, prostacyclin analogs, and milrinone. Sildenafil has also been used extensively in pediatric patients in both the intravenous and the enteral forms in the ICU setting. It may have a synergistic effect when used in combination with iNO or other inhaled agents [23,24,25].

If other vasodilators, for example milrinone, are being used concomitantly with Sildenafil, special attention should be given to avoiding systemic hypotension. As a note, once the acute phase of crisis is resolved, but a patient is not able to tolerate removal of inhaled nitric oxide, sometimes sildenafil can be used as a bridge to be able to wean the nitric oxide and avoid rebound pulmonary hypertension [26]. Intravenous prostacyclin analogs include epoprostenol and treprostinil (which is also used subcutaneously). These agents must be carefully titrated and administered in dedicated lines to avoid interruptions or boluses in doses [27].

##### Support of the Right Ventricle and Systemic Perfusion: The Importance of Preload

Fluid management for this group of patients can be tricky. It can be difficult to find the right balance. IPAH patients have an elevated right atrial pressure and require higher preloads to maintain filling and forward flow. The principles of fluid management include making gentle, small changes if possible, to avoid major fluid shifts. The fluids given for resuscitation should be given judiciously, and the diuresis should be gentle. This becomes even more complex if the patient’s right ventricular function is not preserved. One may consider earlier use of inotropes in this group of patients, to avoid giving huge amounts of intravenous fluids quickly to maintain cardiac output [4].

##### Vasoactive Support

Vasoactive support of both ventricles is indicated in a pulmonary hypertensive crisis. Initially, especially in a hypotensive patient, the first goal of the vasoactive support is to increase the systemic blood pressure—beyond the obvious increase in organ perfusion pressure, the reason is twofold. First, increasing the systemic blood pressure in relation to the pulmonary arterial pressure will reduce the septal shift—allowing the left ventricle to fill and contract more effectively. The second reason to prioritize this rise is to minimize coronary compromise by increasing diastolic blood pressure to overcome the increased end diastolic filling pressures. Unless the left ventricle is experiencing systolic failure—the goal in fact is to increase left ventricular afterload—thus an agent such as norepinephrine or vasopressin is often appropriate [4]. There is some evidence that vasopressin infusion may also lower the PVR in addition to increasing systemic vascular resistance (SVR) [28]. The second goal of vasoactive support is to improve contractility and lusitropy. Additionally, supporting the contraction of the left ventricle will assist the right ventricle. To this end, epinephrine or dopamine infusion may be beneficial. Milrinone infusion in the absence of systemic hypotension, may also be beneficial—and there is also some varying evidence that it may reduce pulmonary vascular resistance as well [29,30].

The perfect medication to treat a pulmonary hypertensive crisis would be one that vasodilates the pulmonary bed, does not cause systemic hypotension by overvasodilating the systemic vascular bed, improves right ventricular function and improves cardiac output. Since there is not one perfect medication to treat pulmonary hypertension, these represent the overriding goals when one chooses a combination of therapies for each patient.

#### 4.4.5. Rescue Therapy

Rescue therapy for patients who are unresponsive to medical therapy may include cannulation to extracorporeal membrane oxygenation. A prerequisite for this risky and resource heavy therapy should be a patient who has foreseeable recovery or alternative option (e.g., transplant). Other rescue options may include surgical/catheter creation of an atrial septal defect or an aortopulmonary shunt creation (Pott’s shunt) [4]. More elaborate discussion of these options is beyond the scope of this article.

## 5. Conclusions

When one thinks about this case, it becomes clear that the entire team’s effort, beginning in the preoperative period, should be geared toward avoiding a pulmonary hypertensive crisis. Once the physiology of a pulmonary hypertensive crisis comes into play, it can be incredibly difficult to reverse, and ultimately can lead to the patient’s demise. When there is a sudden increase in pulmonary arterial pressure, it can be accompanied by a rise in central venous pressure, a decrease in systemic mean arterial pressure, and a decrease in systemic oxygen saturation—low cardiac output ensues. The pulmonary hypertensive crisis represents an often extremely rapid downward spiral for the patient. Recognizing the risk factors for a patient to develop a pulmonary hypertensive crisis and trying to prevent those from occurring is of paramount importance. Once a pulmonary hypertensive crisis occurs, rapid recognition and aggressive treatment is imperative.
